# Pharmacosimulation of delays and interruptions during administration of tirofiban: a systematic comparison between EU and US dosage regimens

**DOI:** 10.1007/s11239-022-02654-0

**Published:** 2022-04-28

**Authors:** Nadia Heramvand, Maryna Masyuk, Johanna M. Muessig, Amir M. Nia, Athanasios Karathanos, Amin Polzin, Marco Valgimigli, Paul A. Gurbel, Udaya S. Tantry, Malte Kelm, Christian Jung

**Affiliations:** 1 Department of Medicine, Division of Cardiology, Pulmonary Diseases and Vascular Medicine, University Hospital Düsseldorf, Moorenstraße 5, 40225 Düsseldorf, Germany; 2 CARID: Cardiovascular Research Institute Düsseldorf, Düsseldorf, Germany; 3 Cardiocentro Ticino, Lugano and University of Bern, Bern, Switzerland; 4 Sinai Center for Thrombosis Research and Drug Development, Sinai Hospital of Baltimore, Baltimore, MD USA

**Keywords:** Tirofiban, GP IIb/IIIa inhibitor, Pharmacokinetics, Pharmacosimulation

## Abstract

Tirofiban is a glycoproteine (GP) IIb/IIIa receptor antagonist, which inhibits platelet-platelet aggregation and is a potential adjunctive antithrombotic treatment in patients with acute coronary syndromes (ACS) or high-risk percutaneous coronary interventions (PCI). It is administered intravenously as a bolus followed by continuous infusion. However, the dosage recommendations in the United States (US) and European Union (EU) differ considerably. Furthermore, in routine clinical practice, deviations from the recommendations may occur. The objective of the present study was to investigate the impact of different alterations on tirofiban plasma concentrations in US and EU administration regimens and to give suggestions for delay management in clinical practice. We therefore mathematically simulated the effects of different bolus-infusion delays and infusion interruptions in different scenarios according to the renal function. Here, we provide a systematic assessment of concentration patterns of tirofiban in the US versus EU dosage regimens. We show that differences between the two regimens have important effects on plasma drug levels. Furthermore, we demonstrate that deviations from the proper administration mode affect the concentration of tirofiban. Additionally, we calculated the optimal dosage of a second bolus to rapidly restore the initial concentration without causing overdosage. In conclusion, differences in tirofiban dosing regimens between the U.S and EU and potential infusion interruptions have important effects on drug levels that may impact on degrees of platelet inhibition and thus antithrombotic effects. Thus, the findings of our modelling studies may help to explain differences in clinical outcomes observed in previous clinical trials on tirofiban.

**Supplementary Information:**

The online version contains supplementary material available at 10.1007/s11239-022-02654-0.

## Highlights

- Tirofiban is a potential adjunctive antithrombotic treatment in patients with ACS or high-risk PCI.
- Maintenance of a proper plasma concentration is crucial for sufficient antithrombotic effect and a better clinical outcome.
- The dosage recommendations for normal or impaired renal function differ significantly between U.S. and EU, which has important effects on plasma drug levels.
- Here, we provide first suggestions for management of delays or interruptions in daily clinical practice, which should be investigated in future studies.

## Introduction

Platelet-fibrinogen interaction is a crucial pathway in platelet aggregation and the pathogenesis of coronary artery thrombosis [1]. By binding to glycoprotein (GP) IIb/IIIa receptors, fibrinogen ensures platelet-platelet aggregation and thrombus formation at the site of vascular injury [2]. Thus, blockade of GP IIb/IIIa receptor is a potential additional antithrombotic treatment strategy [3, 4]. Tirofiban is an intravenous non-peptide reversible GP IIb/IIIa receptor antagonist [5, 6]. Several large randomized controlled trials (RCTs) have shown the antithrombotic benefits of tirofiban use in patients with acute coronary syndromes (ACS) and in high-risk percutaneous coronary interventions (PCI) [6–9]. A PCI is considered as a high-risk procedure when several characteristics, including complex coronary artery disease (multivessel or left main disease and anatomically complex coronary lesions), hemodynamic compromise (shock or severely depressed LV function), and clinical comorbidities such as advanced age, diabetes mellitus, peripheral vascular disease, heart failure, acute coronary syndromes, or previous cardiac surgery, apply [10]. A meta-analysis including 6 large RCTs with 29,570 non ST-elevation ACS patients has confirmed a significant reduction of 30-day mortality or non-fatal myocardial infarction (MI) in patients receiving GP IIb/IIIa inhibitors [11]. However, bleeding events remain a major concern [6]. Thus, current European Society of Cardiology (ESC) and American Heart Association/American College of Cardiology (AHA/ACC) guidelines recommend the use of GP IIb/IIIa inhibitors in ACS patients treated invasively with dual antiplatelet therapy (DAPT) with a Class IIb for bailout situations or thrombotic complications during PCI [12, 13]. Tirofiban is administered intravenously as a bolus followed immediately by continuous infusion. The current dosage regimens are regulated and approved by the United States (US) Food and Drug Administration (FDA) and European Medicines Agency (EMA). However, the dosage recommendations of the two regulatory bodies differ considerably [14, 15].

In routine clinical practice, several deviations from the recommendations, such as delay between the bolus and the infusion or infusion interruptions may occur. The purpose of the present study was to investigate, by mathematical modelling, the impact of various deviations from the recommended tirofiban administration regimen on plasma concentrations for both US and EU tirofiban on-label regimens and provide practical suggestions for their optimal pharmacological management.

## Methods

Simulations were performed using the Python™ programming language. First, a two-compartment pharmacokinetic (PK) model of tirofiban has been applied, as after a single intravenous bolus, the plasma concentration–time of tirofiban passes through distribution and disposition phases (Supplementary Fig. 1 a). Therefore, the plasma concentration–time of tirofiban could be described through *Cp(t)* = *Ae − αt* + *Be*^*–βt*^ equation, where *Cp(t)* is the plasma concentration at any time (t), *A* and *B* are empirical constants, *α* and *β* are distribution and disposition rate constants, respectively. This biphasic behaviour of plasma concentration could be also explained as tirofiban is not strongly bound to plasma protein with an unbound fraction in human plasma of 35%[14]. In the next step, *A*, *B*, *α* and *β* were estimated by applying the method of residuals [16] and based on real-world concentration measurements at different time points in patients with normal or impaired renal function, obtained during product development (Supplementary Table 1). Briefly, patients were treated with a 25 μg/kg tirofiban bolus. The patients were subdivided into three groups according to the renal function: patients with normal function (creatinine clearance; CrCl > 90 ml/min; *n* = 8), moderate (CrCl 30–59 ml/min; *n* = 8) and severe renal impairment (CrCl < 30 ml/min; *n* = 7). Patients with mild renal impairment (CrCl 90–60 ml/min) were not included in the dataset. Plasma concentrations of tirofiban were measured after 0.25, 0.50, 0.75, 1, 2, 3, 4, and 6 h in all patients and additionally after 8, 10, and 12 h in patients with renal impairment. Supplementary Table 2 presents the estimated PK parameters.

The elimination of tirofiban occurs by renal and biliary excretion, as it has been shown by experiments with radioactively labelled tirofiban administered to healthy individuals. Here, 66% of radioactivity was recovered in the urine and 23% in the feces, with a total recovery of radioactivity of 91%. The half-life of tirofiban is approximately 1.5 h. In clinical studies, patients with decreased renal function showed a reduced plasma clearance of tirofiban. Thus, in patients with creatinine clearance < 30 ml/min, the plasma clearance is reduced over 50%. [15]. The second term of the previous equation, *Be*^*–βt*^, reflects the elimination of the tirofiban from the body. For renally impaired subjects, *Be*^*–βt*^ declines slower, indicating that the elimination half-life of tirofiban is greater, which results in a higher concentration of the drug, compared with subjects with a normal renal function. Then, to simulate the time profile of plasma concentrations of tirofiban for different scenarios, a two-compartment ordinary differential equation (ODEs) was applied:

$$\begin{gathered} \frac{dXc}{{dt}} = I(t) + k_{21} X_{p} - k_{12} X_{c} - k_{10} X_{c} \hfill \\ \frac{dXc}{{dt}} = k_{12} X_{C} - k_{21} X_{p} \hfill \\ \end{gathered}$$,

where.

*I*(*t*) is the rate of drug administration and has units of mass.time^−1^, *X*_*c*_ is the amount of drug in the central compartment and has units of mass, *X*_*p*_ is the amount of drug in the peripheral compartment and has units of mass, *k*_12_ is the first-order transfer rate constant from the central compartment to the peripheral compartment and has units of time^−1^, *k*_21_ is the first-order transfer rate constant from the peripheral compartment to the central compartment and has units of time^−1^, *k*_10_ is the first-order elimination rate constant from the central compartment and has units of time^−1^.

Here the estimated α, β, A and B were used to calculate the ODEs parameters [16].The ODE set was integrated using the “solve_ivp” function from the SciPy package (version 1.1.0). By solving the two sets of ODEs with different conditions, the effect of different delays and interruptions was evaluated. Finally, the dosage of a second bolus was estimated allowing a rapid recovery of tirofiban plasma levels within the anticipated therapeutic window. This optimization was performed using “Nelder-Mead” method of the “minimize” function from the SciPy package (version 1.1.0).

## Results

### Patient characteristics

The real-world cohort during the product development consisted of overall 23 patients (normal renal function: *n* = 8, moderate impairment: *n* = 8, severe impairment: *n* = 7). Patients were of a mean age of 60.4 years (range 31–82 years). Patients in the normal renal function group had a slightly higher weight (90.9 vs. 82.0 kg) and BMI (31.46 vs. 28.60 kg/m^2^) compared to those in the moderate and severe renal function groups. Patients were almost equally split between male and female (11 male vs. 12 female) with more White patients (17 patients) enrolled compared to Black/African American (6 patients). There were no patients of Hispanic/Latino ethnicity or of Asian descent. None of the patients had acute coronary syndrome.

### Parameter estimation and model verification

PK parameters of tirofiban for the three groups of patients according to renal function have been estimated as described above (Supplementary Table 2). Calculated PK parameter estimates were used to develop a dynamic model to simulate the plasma concentration–time profile of tirofiban. Here, we demonstrate a good fit between modelled and real-world patient data, thus confirming our model to be suitable for further simulations (Supplementary Fig. 1b–d).

### Comparison between EU and US dosage regimens

**Fig. 1:**
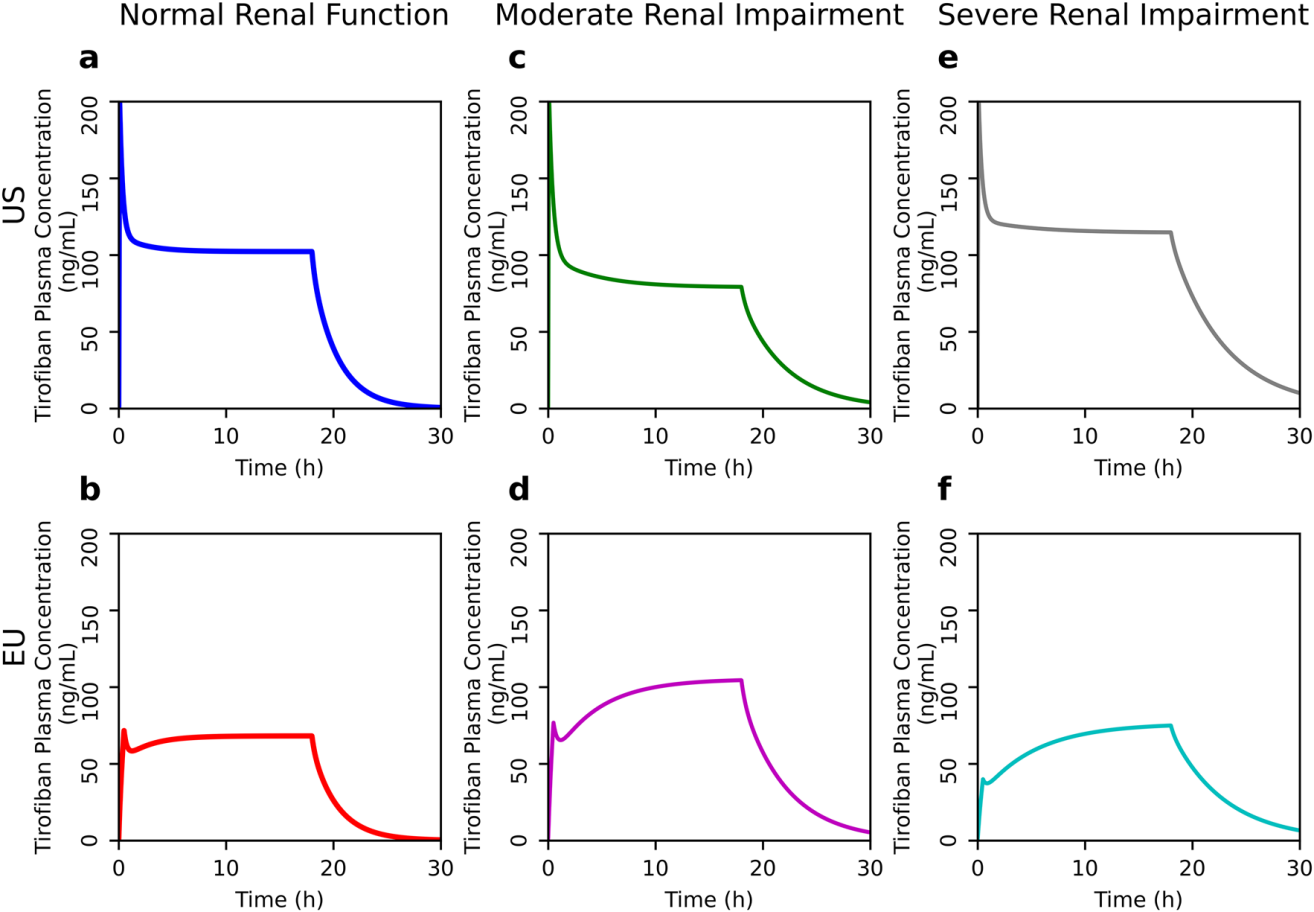
Plasma concentration–time curves of tirofiban according to renal function in US **(a, c, e)** and EU **(b, d, f)** dosing regimens

Based on calculated PK parameters, simulations were carried out by applying EU and US dosage recommendations, as described above. Briefly, EU recommendations implicate both a lower loading and maintenance doses (loading concentration of 0.4 µg/kg/min over 30 min, followed by 0.1 µg/kg/min infusion) than US recommendations (25 μg/kg within 5 min followed by infusion at a rate of 0.15 μg/kg/min for up to 18 h) in patients with normal renal function. Furthermore, renal dose adjustment is recommended only for patients with severe renal impairment (CrCl < 30 ml/min) in EU but start with moderately impaired function (CrCl ≤ 60 ml/min) in the US However, while EU recommends reduction of both, bolus and infusion rate, US recommendations include an unchanged bolus dose followed by a reduced maintenance infusion rate [14, 15]. Figure 1 shows plasma concentration–time curves of tirofiban for three groups of patients according to renal function. In all scenarios, in the US regimen, the initial plasma concentration following bolus administration is considerably higher and the steady-state concentration is achieved faster than in the EU regimen. In case of normal renal function as well as severe renal impairment, where the dosage reduction occurs in both regimens, the steady-state plasma concentration is lower in the EU than in the US regimen. However, in severe renal impairment, the steady-state concentration in the EU regimen is reached even more slowly than in normal function. For moderate renal function, the steady-state concentration is higher in the EU than in the US regimen.

### Effects of delays and interruptions in EU and US dosage regimens

**Fig. 2:**
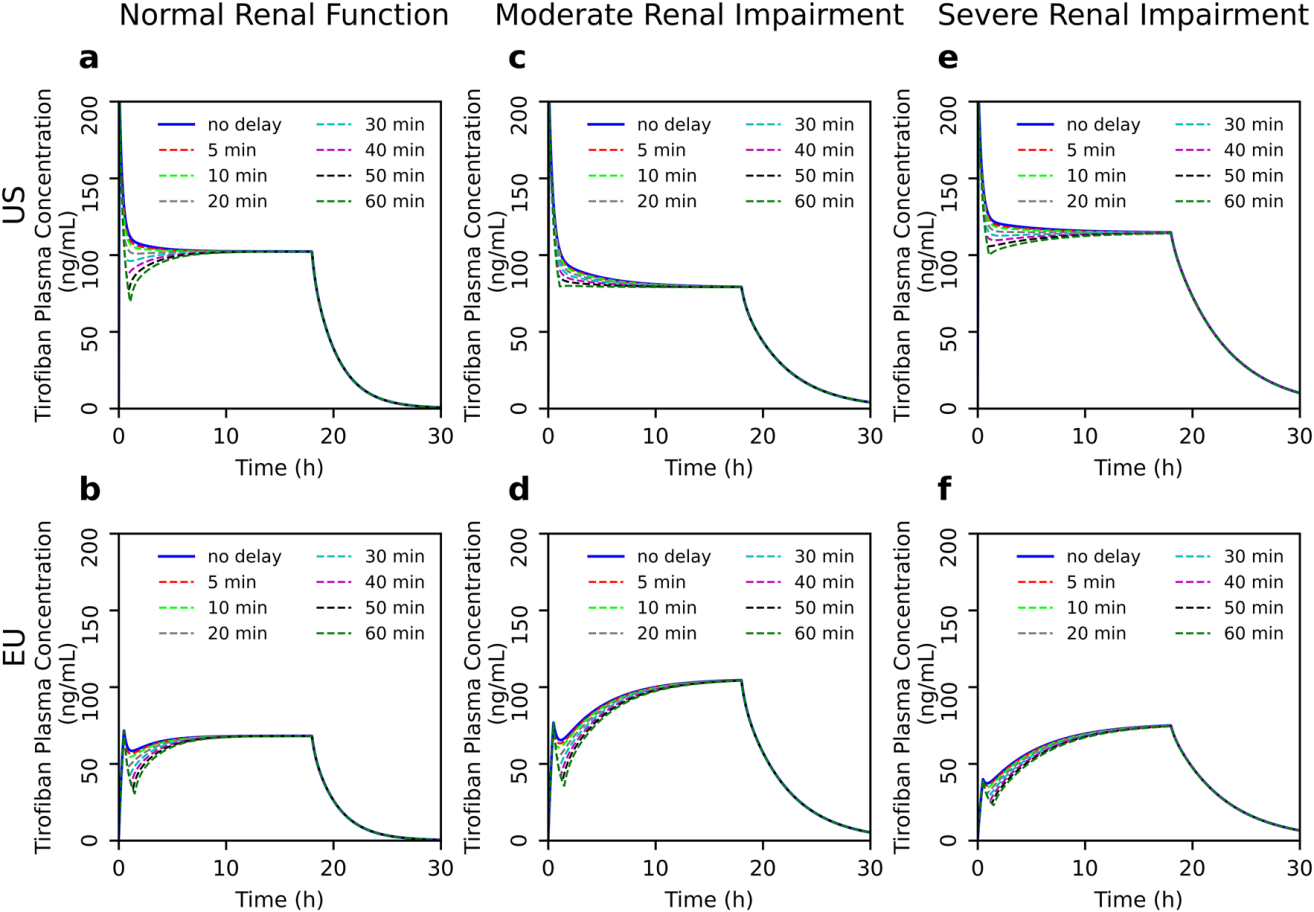
Effects of different delays between bolus and initiation of continuous infusion of tirofiban on plasma drug concentration in different populations according to renal function. Comparison between US **(a, c, e)** and EU **(b, d, f)** dosing regimens

**Fig. 3:**
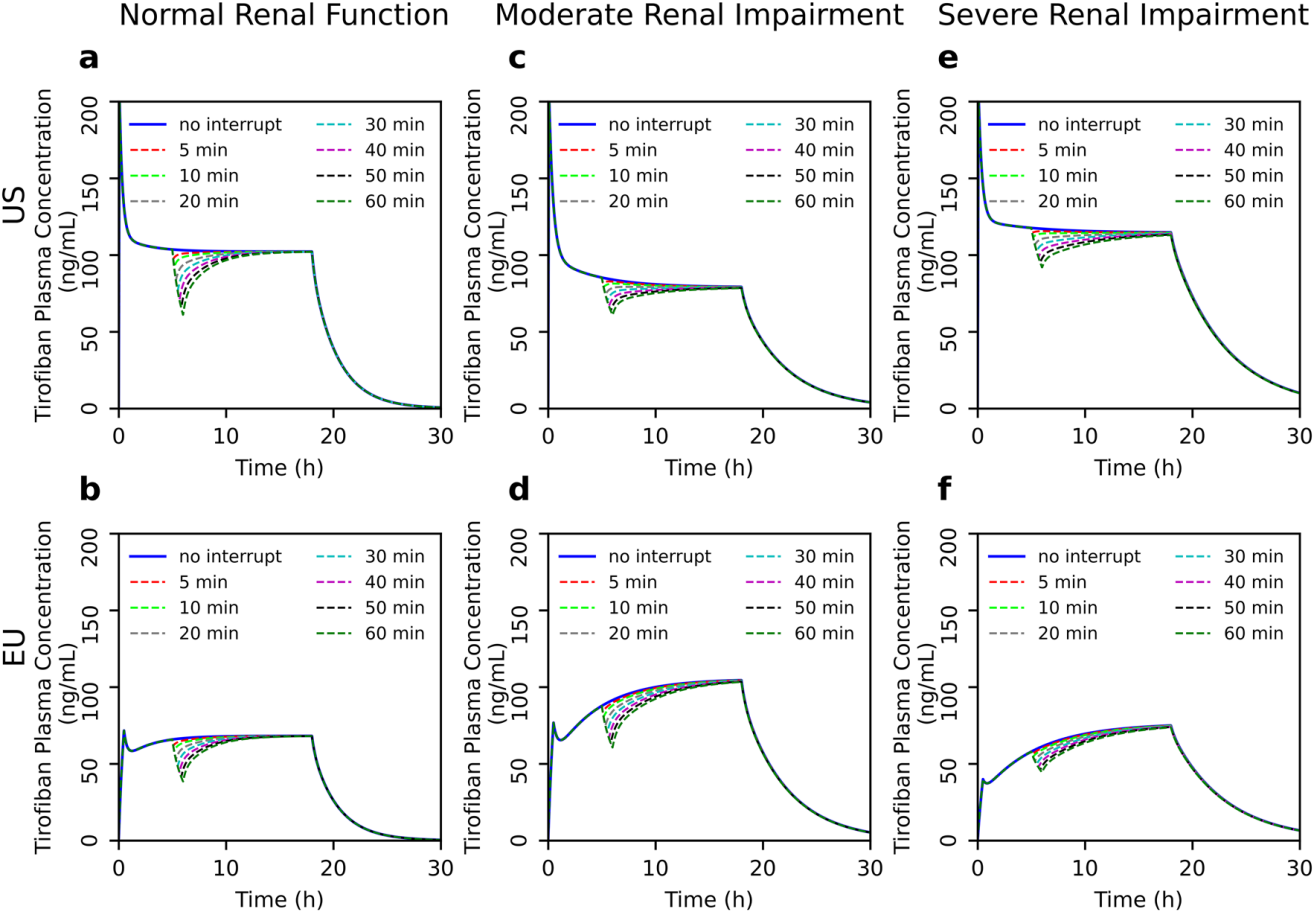
Effects of different interruptions of continuous infusion of tirofiban on plasma drug concentration in different populations according to renal function. Comparison between US **(a, c, e)** and EU **(b, d, f)** dosing regimens

In the next step, we mathematically simulated the effect of different delays and interruptions of administration on plasma drug concentration. Here, we demonstrate that short delays between the bolus and initiation of continuous infusion do not result in significant changes in the drug concentrations in either EU or US regimens regardless of renal function (Fig. 2). However, a longer than 30-min delay leads to considerable decrease in plasma concentrations in the EU dosing regimen in all three groups (Fig. 2b, d, f). Notably, in the US regimen, the concentration decrease after delays of more than 30 min is lower than in EU dosing. Furthermore, in moderate or severe renal dysfunction group, the influence of delay is less pronounced than in normal renal function in US regimen (Fig. 2a, c, e). Similarly, we show that interruptions of continuous infusion over 30 min lead to considerable decrease in tirofiban plasma concentrations (Fig. 3). This effect is even more pronounced in the EU regimen (Fig. 3 b, d, f). According to the US dosage recommendations, the decrease in concentrations after interruption are less pronounced in renal dysfunction group than in case of normal renal function group (Fig. 3a, c, e).

Additionally, we estimated the dosage of a possible second bolus to compensate for the delays or interruptions in intravenous tirofiban application in both EU and US regimens (Table 1 and 2; Supplementary Fig. 2 and 3).

**Table 1:** Dosage of the 2nd bolus after different delays between bolus administration and initiation of infusion of tirofiban in patients with normal renal function

	Delay (min)	5	10	20	30	40	50	60	70
US	Dosage of 2nd bolus (µg/kg)	–	–	–	1.5	2.84	4.05	5.15	6.16
	in % of 1st bolus	–	–	–	6%	11%	16%	21%	25%
EU	Dosage of 2nd bolus (µg/kg)	0.58	0.64	0.76	0.86	0.95	1.04	1.11	1.19
	in % of 1st bolus	5%	5%	6%	7%	8%	9%	9%	10%

**Table 2:** Dosage of the 2nd bolus after different infusion interruptions of tirofiban in patients with normal renal function

	Interruption (min)	5	10	20	30	40	50	60	70
US	Dosage of 2nd bolus (µg/kg)	0.48	1.18	2.5	3.7	4.82	5.86	6.83	7.73
	in % of 1st bolus	2%	5%	10%	15%	19%	23%	27%	31%
EU	Dosage of 2nd bolus (µg/kg)	0.16	0.24	0.37	0.5	0.61	0.72	0.82	0.92
	in % of 1st bolus	1%	2%	3%	4%	5%	6%	7%	8%

## Discussion

Several studies have investigated the effect of the GP IIb/IIIa inhibitor tirofiban in patients with ACS or in patients undergoing high-risk PCI. The PRISM-PLUS trial showed a reduction in major adverse cardiac events (MACE) at 30 days with GP IIb/IIIa inhibitor plus unfractionated heparin as compared to unfractionated heparin alone [7]. In the Randomized Efficacy Study of Tirofiban for Outcomes and Restenosis (RESTORE) trial, the administration of tirofiban was not associated with a significant reduction in MACE [8]. A study by Steinhubl et al. including 501 patients treated with abciximab, tirofiban and eptifibatide, demonstrated that the levels of platelet function inhibition are independently associated with the rate of MACE after PCI [17]. Moreover, it has been demonstrated that normal myocardial perfusion after fibrinolytic therapy for ST-elevation myocardial infarction (STEMI) and after PCI for ACS is associated with higher GPI receptor occupancy in the setting of eptifibatide therapy [18–20]. Thus, higher doses of tirofiban would similarly be expected, in turn, to provide greater receptor occupancy to provide sufficient platelet inhibition to translate into a beneficial clinical effect. In 2004, the ADVANCE trial demonstrated a significant reduction of ischemic events using tirofiban in the setting of high-risk PCI when administered at a high dose bolus of 25 μg/kg followed by infusion of 0.15 μg/kg/min for 24–48 h [9]. A systematic pooled meta-analysis of RCTs investigating tirofiban versus placebo or abciximab including over 20,000 patients confirmed a reduction of death or combined endpoint of death and MI with the use of tirofiban. [21].

All these studies strengthen our hypothesis that maintenance of a proper plasma concentration of tirofiban is crucial for sufficient antithrombotic effect and a better clinical outcome. Interestingly, the dosage recommendations for tirofiban differ between EU and US In the present study, we systematically compare two dosing regimens and simulated different deviations from the recommended administration mode using a mathematical model. USUSOur simulations of tirofiban plasma concentration in normal renal function, using the US dosing regimen, demonstrated a faster increase in plasma concentration to almost double steady-state level followed by a drop to a still high level of plasma tirofiban concentration of over 100 ng/ml. In the EU dosing regimen, the bolus is administered at a lower dose over a longer timeframe followed by continuous infusion. This leads to almost constant plasma tirofiban levels during the entire administration period, which is, however, considerably lower than in the US regimen. By contrast, in case of moderate renal impairment, the steady-state concentration in the EU regimen is higher than in the US regimen as there is no dosage adjustment in the EU recommendation. In our model of severe renal insufficiency, we have demonstrated a lower steady-state tirofiban concentration, which is reached more slowly in the EU than in the US regimen. This can be explained by the fact that in the EU regimen, both the loading as well as maintenance doses are reduced, while the bolus dose remains unchanged in the US recommendations. Furthermore, we show here that deviations from the proper administration mode affect the concentration of tirofiban. However, shorter delays or interruptions do not have a major impact on plasma drug levels, whereas deviations of over 30 min show considerable effects. This finding is consistent with the elimination half-life of tirofiban of approximately 2 h [6]. Of note, regardless of renal function, the changes in plasma levels are less pronounced using the US regimen compared to the EU regimen. This is most likely due to a higher dose of initial bolus in the US regimen, which is not reduced even in case of severe renal impairment.

In the context of clinical studies, the EU regimen in patients with normal renal function is comparable to the dosage used in the PRISM-PLUS trial [7], whereas the US dosing regimen was used in the ADVANCE trial [9], both showing a beneficial antithrombotic effect of tirofiban. By contrast, in the RESTORE trial which failed to demonstrate a MACE reduction by use of GP IIb/IIIa inhibitors, a lower bolus dose has been applied (10 μg/kg bolus followed by infusion at a rate of 0.15 μg/kg/min). Therefore, one could speculate that both EU and US regimens are equal in terms of their antithrombotic effect. Despite the higher steady-state drug level in the US dosing regimen, the ADVANCE study did not reveal a higher rate of adverse events such as major bleedings [9]. Nonetheless, it is important to note that the present study is based on a simulated mathematical model. Even though we demonstrated that the changes in drug concentration following delays or interruptions in the US regimen are less pronounced than in the EU regimen, it is not clear what concentration is required to induce adequate effect on platelet aggregation. This represents the main limitation of our study as we only provide a pharmacokinetics simulation without taking into account the pharmacodynamics of the drug. Another pharmacokinetic modelling study published by Lakings et al. in 2012 used the simulation approach to identify an appropriate dosage in patients with severely impaired renal function which would lead to a similar tirofiban time-concentration profile as reached by the US regimen dosage in patients with normal renal function [22]. When comparing the real-life and modeled concentration–time profiles in this study and our study at the same dosing regimens, it is notable that in our study the steady-state plasma levels are considerably higher in both normal and severely impaired renal function. Even more pronounced is the concentration difference in patients with severe renal impairment after the recommended dosing rate adjustment. This is surprising as the reduced renal elimination would expectedly lead to higher plasma concentrations, as it is the case in our simulations. However, the differences in the estimated PK parameters in both studies are most likely due to a relatively low number of measurements, on which the estimated parameters are based. This is another limitation of the present study.

In conclusion, differences in tirofiban dosing regimens between the US and EU and potential infusion interruptions have important effects on drug levels that may impact, in turn, on degrees of platelet inhibition. The totality of evidence supports that high levels of receptor occupancy by GPIs are required to reduce clinical thrombotic events. Thus, the findings of our modelling studies may help to explain differences in clinical outcomes observed in trials of tirofiban for the treatment of high-risk coronary artery disease. Our data indicates towards equality of both regimens in terms of clinical outcomes and possible higher probability of side effects due to a higher steady-state concentration in US regimen. However, this study remains a mathematical model and evidently future clinical trials are required for a real-world comparison between the two regimens in different clinical settings.

## Supplementary Information

Below is the link to the electronic supplementary material.

Supplementary file1 (TIFF 11829 KB)

Supplementary file2 (TIFF 14104 KB)

Supplementary file3 (TIFF 14431 KB)

## Data Availability

The data underlying this article are available in the article and in its online supplementary material.

## Code availability

Python™ codes to simulate pharmacokinetics of tirofiban in this manuscript are available on request.

## References

[1] Fuster V, Badimon L, Badimon JJ (1992). The pathogenesis of coronary artery disease and the acute coronary syndromes (1). N Engl J Med.

[2] van de Werf F (1997). Clinical trials with glycoprotein IIb/IIIa receptor antagonists in acute coronary syndromes. Thromb Haemost.

[3] Coller BS (1995). Blockade of platelet GPIIb/IIIa receptors as an antithrombotic strategy. Circulation.

[4] Lefkovits J, Plow EF, Topol EJ (1995). Platelet glycoprotein IIb/IIIa receptors in cardiovascular medicine. N Engl J Med.

[5] Hartman GD, Egbertson MS, Halczenko W (1992). Non-peptide fibrinogen receptor antagonists. 1. Discovery and design of exosite inhibitors. J Med Chem.

[6] McClellan KJ, Goa KL (1998). Tirofiban. A review of its use in acute coronary syndromes. Drugs.

[7] Platelet Receptor Inhibition in Ischemic Syndrome Management in Patients Limited by Unstable Signs and Symptoms (PRISM-PLUS) Study Investigators (1998). Inhibition of the platelet glycoprotein IIb/IIIa receptor with tirofiban in unstable angina and non-Q-wave myocardial infarction. N Engl J Med.

[8] Investigators R (1997). Effects of platelet glycoprotein IIb/IIIa blockade with tirofiban on adverse cardiac events in patients with unstable angina or acute myocardial infarction undergoing coronary angioplasty. The RESTORE Investigators. Randomized Efficacy Study of Tirofiban for Outcomes and REstenosis. Circulation.

[9] Valgimigli M, Percoco G, Barbieri D (2004). The additive value of tirofiban administered with the high-dose bolus in the prevention of ischemic complications during high-risk coronary angioplasty: the ADVANCE trial. J Am Coll Cardiol.

[10] Bass TA (2015). High-risk percutaneous coronary interventions in modern day clinical practice: current concepts and challenges. Circ Cardiovasc Interv.

[11] Roffi M, Chew DP, Mukherjee D (2002). Platelet glycoprotein IIb/IIIa inhibition in acute coronary syndromes. Gradient of benefit related to the revascularization strategy. Eur Heart J.

[12] Amsterdam EA, Wenger NK, Brindis RG (2014). 2014 AHA/ACC guideline for the management of patients with non-ST-elevation acute coronary syndromes: a report of the American College of Cardiology/American Heart Association Task Force on Practice Guidelines. J Am Coll Cardiol.

[13] Roffi M, Patrono C, Collet J-P (2016). 2015 ESC Guidelines for the management of acute coronary syndromes in patients presenting without persistent ST-segment elevation: task force for the management of acute coronary syndromes in patients presenting without persistent ST-segment elevation of the European Society of Cardiology (ESC). Eur Heart J.

[14] U.S. Food and Drug Administration (2001) Aggrastat (tirofiban Hydrochloride) injection & premixed injection: drug approval package. https://www.accessdata.fda.gov/drugsatfda_docs/nda/99/20912S001_Aggrastat.cfm. Accessed January 2020.

[15] European Medicines Agency. The European Agency for the Evaluation of Medicinal Products (1999) Opinion following an article 10 referral Aggrastat. International Nonproprietary Name (INN): Tirofiban. https://www.ema.europa.eu/en/documents/referral/opinion-following-article-10-referral-aggrastat-international-non-proprietary-name-inn-tirofiban_en.pdf. Accessed March 2020

[16] Jambhekar SS, Breen PJ (2009). Basic pharmacokinetics.

[17] Steinhubl SR, Talley JD, Braden GA (2001). Point-of-care measured platelet inhibition correlates with a reduced risk of an adverse cardiac event after percutaneous coronary intervention: results of the GOLD (AU-assessing Ultegra) multicenter study. Circulation.

[18] Gibson CM, Jennings LK, Murphy SA (2004). Association between platelet receptor occupancy after eptifibatide (integrilin) therapy and patency, myocardial perfusion, and ST-segment resolution among patients with ST-segment-elevation myocardial infarction: an INTEGRITI (Integrilin and Tenecteplase in Acute Myocardial Infarction) substudy. Circulation.

[19] Deibele AJ, Jennings LK, Tcheng JE (2010). Intracoronary eptifibatide bolus administration during percutaneous coronary revascularization for acute coronary syndromes with evaluation of platelet glycoprotein IIb/IIIa receptor occupancy and platelet function: the Intracoronary Eptifibatide (ICE) Trial. Circulation.

[20] Gurbel PA, Tantry US (2010). Delivery of glycoprotein IIb/IIIa inhibitor therapy for percutaneous coronary intervention: why not take the intracoronary highway?. Circulation.

[21] Valgimigli M, Biondi-Zoccai G, Tebaldi M (2010). Tirofiban as adjunctive therapy for acute coronary syndromes and percutaneous coronary intervention: a meta-analysis of randomized trials. Eur Heart J.

[22] Lakings DB, Janzen MC, Schneider DJ (2012). Pharmacokinetic modeling of the high-dose bolus regimen of tirofiban in patients with severe renal impairment. Coron Artery Dis.

