# Supplementary figures and images for: Pharmacosimulation of delays and interruptions during administration of tirofiban: a systematic comparison between EU and US dosage regimens
Source: J Thromb Thrombolysis. 2022 Apr 28;54(2):301–8. doi: 10.1007/s11239-022-02654-0 (PMC9363357; doi:10.1007/s11239-022-02654-0)

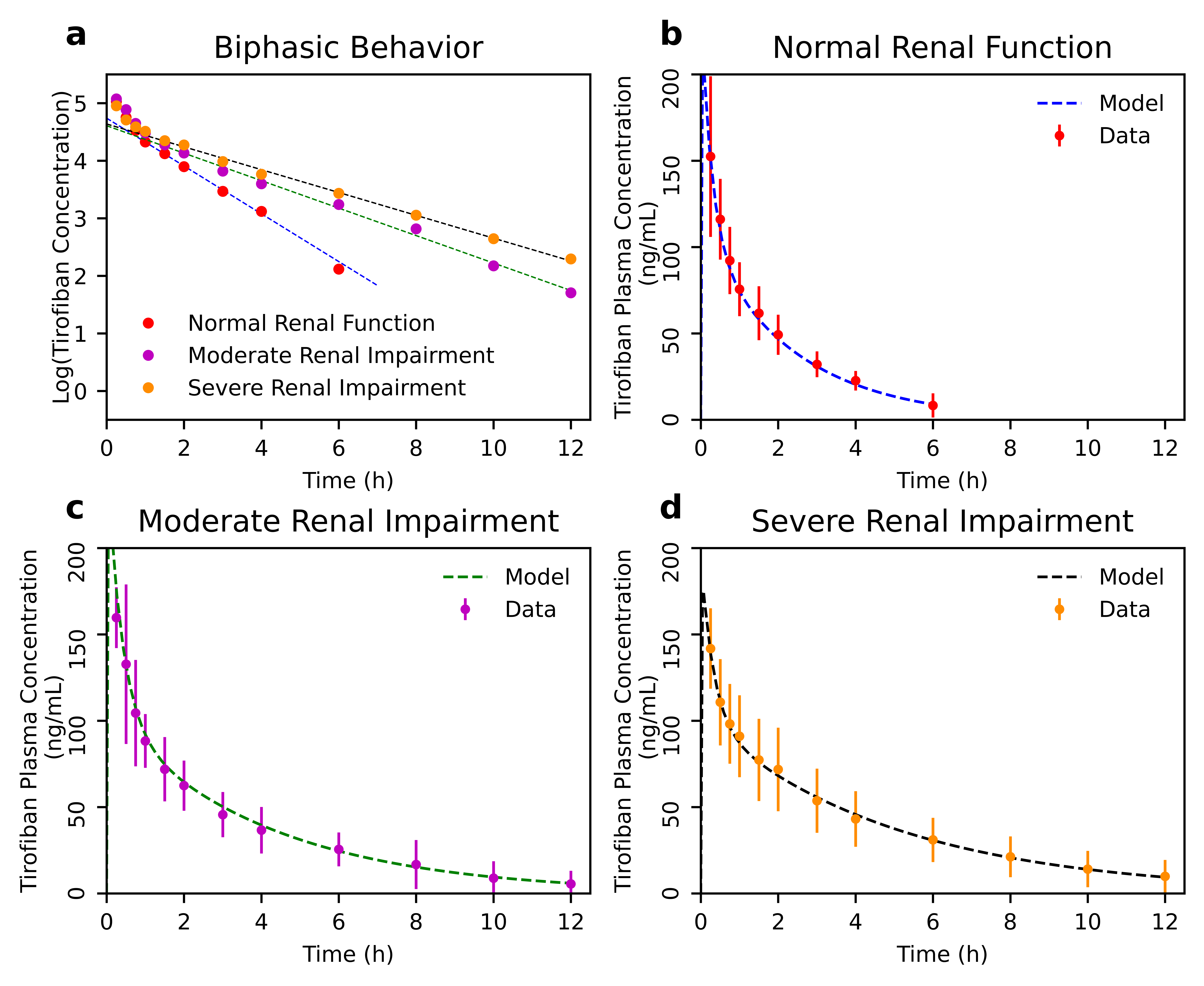

Supplement: Supplementary file 1 — Supplementary file1 (TIFF 11829 KB) [file 11239_2022_2654_MOESM1_ESM.tiff]

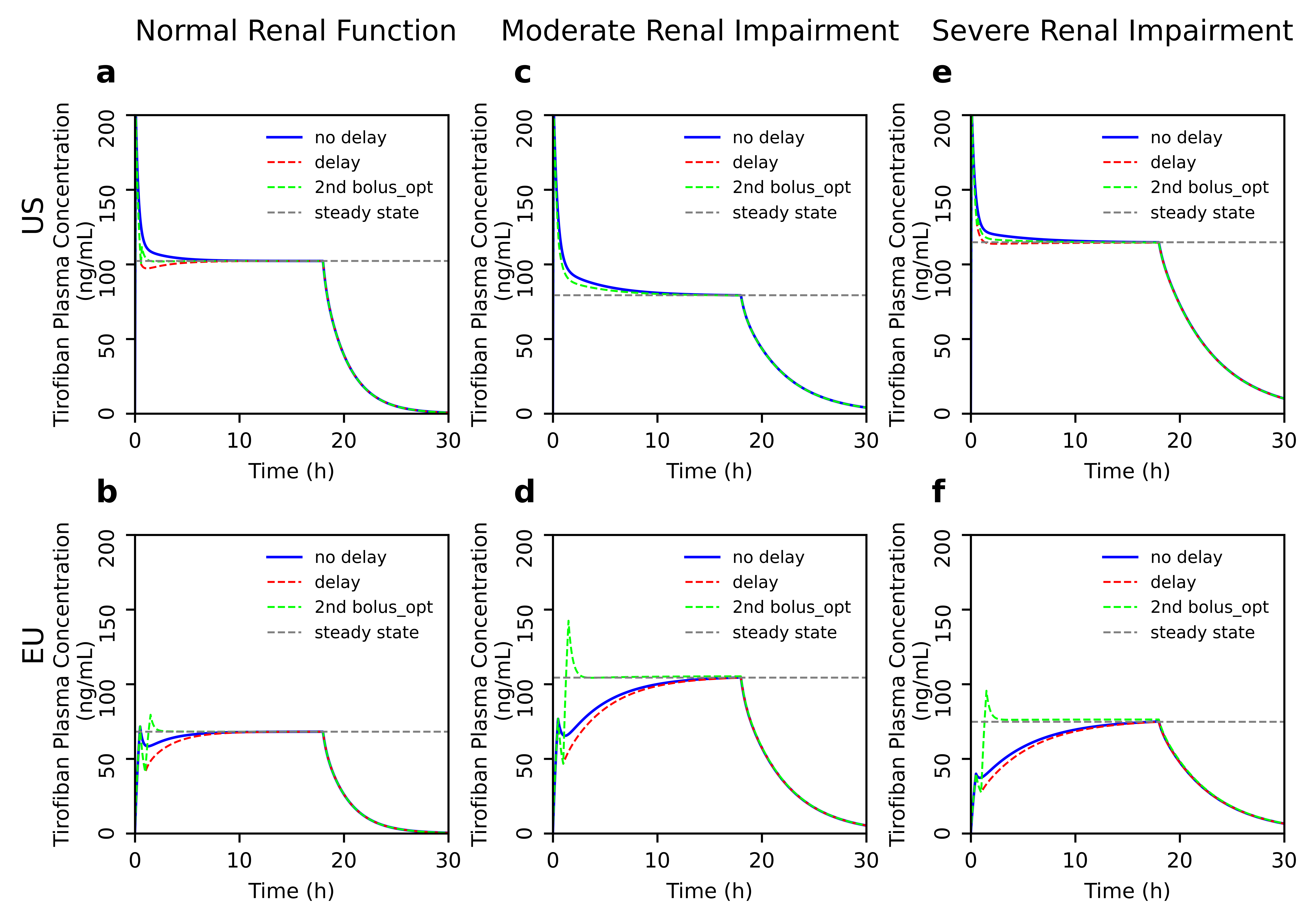

Supplement: Supplementary file 2 — Supplementary file2 (TIFF 14104 KB) [file 11239_2022_2654_MOESM2_ESM.tiff]

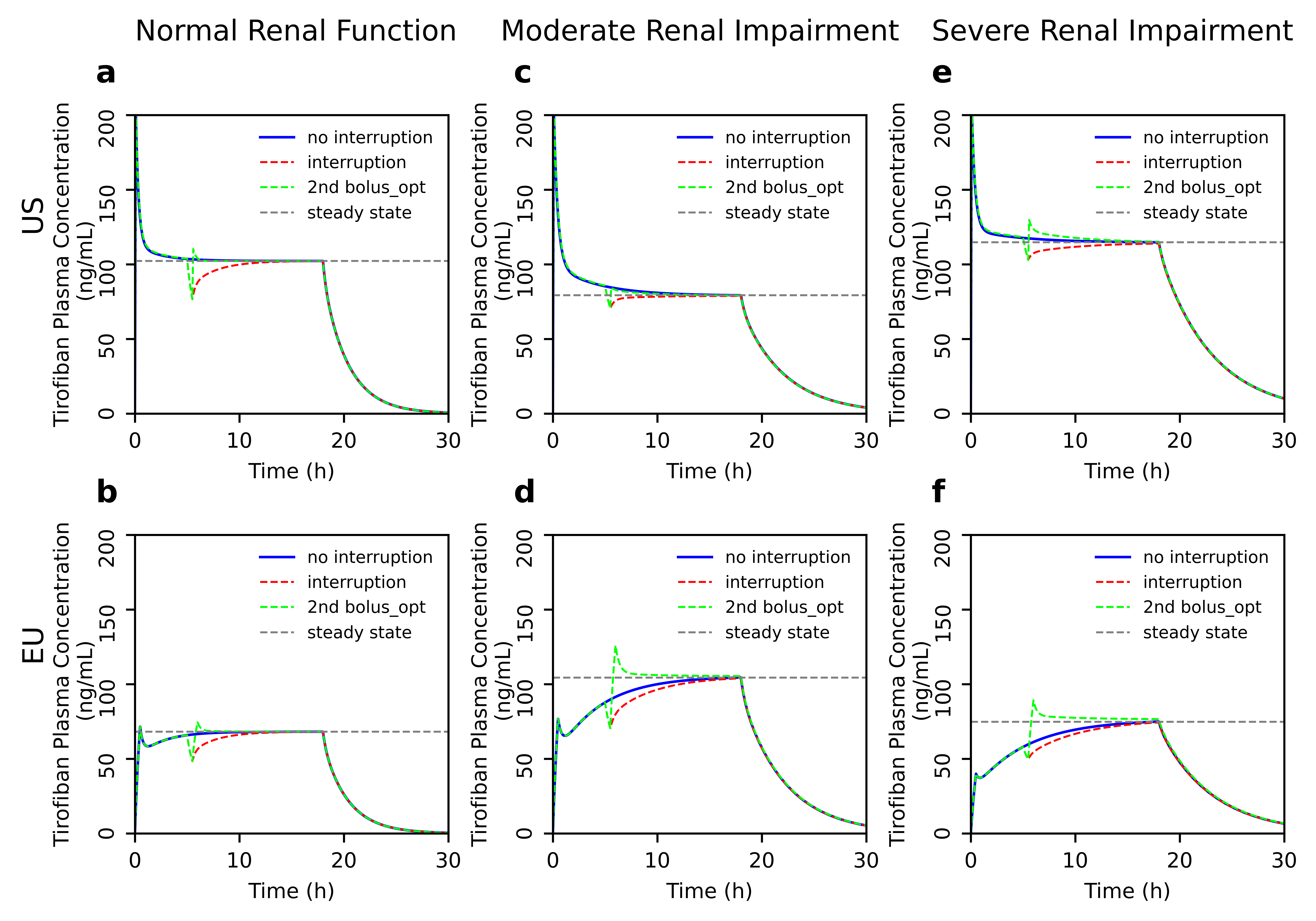

Supplement: Supplementary file 3 — Supplementary file3 (TIFF 14431 KB) [file 11239_2022_2654_MOESM3_ESM.tiff]
